# Correspondence from New York

**Published:** 1865-02

**Authors:** 


### CORRESPONDENCE.

NEW YORK.

The Commissioners of Public Charity of this City have been sending, for the past nine months, all of their patients with typhus fever to Blackwell's Island for treatment. They are there placed in canvas tents, similar to the army hospital tents, for two objects: first, to seclude the cases to prevent the spread of the contagion; and, secondly, to give them plenty of fresh air. Dr. A. L. LOOMIS, of this City, has had medical charge, and treated them entirely with hygienic means—giving especial attention to ventilation. The number of cases continuously under treatment, has averaged seventy, or about six hundred in all. And not a particle of medicine, and no stimulants have been employed, and the result, so far, has been but one death in seventeen cases! This is certainly the most remarkable success ever obtained in the treatment of this disease, and seems almost incredible. But the statement is made on the authority of the Medical Board of Bellevue Hospital, under whose supervision these observations have been made. Your correspondent visited, several times during the winter, these typhus patients, and can testify that a large number of them seemed very dangerously sick, yet their only treatment consisted in giving them air as fresh as the heavens could distil it, with simple nourishment, and the result is certainly marvellous, in our eyes.

The number of cases of typhus fever treated in Bellevue Hospital for three years prior to the removal of this class of patients to Blackwell's Island—from June 1st, 1861, to June 1st, 1864—was nine hundred and eight, of which one hundred and ninety-eight proved fatal, making one death in about four and a-half cases. Excluding those who died within forty-eight hours after their admission, the mortality was about one in six. Dr. MURCHISON, in a report of a large number of typhus treated in the London Fever Hospital, gives a mortality of one in five. Dr. M.'s treatment was the stimulant plan, and so in Bellevue. Dr. LOOMIS was induced to reject alcoholic stimulants from certain views which he entertained relating to the pathology of the disease. He had found, in making autopsies of twenty or thirty of the fatal cases at Bellevue Hospital, that the most constant morbid appearance met with was an intensely congested state of the kidneys. He had also found albumen and casts in the urine during the continuance of the disease, in a large number of cases. From these facts Dr. L. concluded that alcohol was contra-indicated as increasing the burden, and otherwise favoring the congestion of the kidneys. He resolved, therefore, to dispense with it in toto in the treatment of the cases on Blackwell's Island. Whether the very favorable results there obtained are partly due to this fact, or entirely to the hygienic measures, further observations must decide. It is a fact, however, worthy of note, that both Profs. CLARK and FLINT, in their lectures to their respective classes on the treatment of typhus fever, recommended the 'expectant' plan and a resort to stimulants only when great exhaustion supervened. The Profession here are looking with great interest for a paper on this subject, which Dr. LOOMIS has promised to read to the Academy of Medicine at its next meeting.

On visiting the wards of the New York and Bellevue Hospitals, one is struck with the number of cases of lead poisoning which are met with. The attending physicians have no difficulty in tracing the larger number of these cases to the use of beer, the article having been in contact with the lead faucet, or otherwise in lead pipe. It is a sad commentary on this beverage that it is largely chargeable with the damaged livers met with in almost every subject examined in the dead-house connected with these hospitals, but the additional credit of conveying into so many systems the terrible poison of lead. But, thanks to our science, a sure remedy is found. That iodide of potash will eliminate the poison of lead —as well as mercury— from the system, has been demonstrated too often to leave any doubt on the subject. It is *the* remedy *par excellence* which is always prescribed here in these cases, and continued as long as chemical tests reveal the presence of lead in the urine.

The health of New York, at present, is in a deplorable state. Contagious and infectious diseases abound on almost every street. A Committee has canvassed the City and brought to light *fourteen hundred cases* of variola. And the Mayor has issued a proclamation, ordering every one vaccinated, and re-vaccination in every case where the operation had not been performed within five years. Typhus fever, typhoid pneumonia, erysipelas, and diphtheria are also very prevalent. Several physicians of the City are now in Albany, laboring with the Legislature to secure a Sanitary Reform, and are abundantly prepared to prove two facts that stand very much in the relation of cause and effect. First, that New York is the filthiest City on the Continent; and secondly, it is one of the most sickly. Figures prove the latter, and residents familiar with different sections of the City testify to the former. Broadway and Fifth Avenue are justly the pride of the New Yorker, and, it is said, are swept every day. But the vastly larger number of streets know no broom, and are strangers to the visits of the scavenger. If something is not done speedily to cleanse the City a pestilence may justly be feared.

The Profession here are already preparing for a large representation at the next meeting of the American Medical Association, in Boston. The stimulus afforded by the last meeting in this City, has aroused many to labor anew to promote the usefulness of our National Medical Council. It is expected, by many, that so large a "turn out" of "Medicos," the "Hub of the Universe" never witnessed as will meet there in June. These Annual Meetings of the Profession can in no wise interfere with the success of GRANT'S plans in conquering an honorable peace, or with Congress, in building a ship canal! If there ever is a time when medical men should meet for mutual conference, it is during this time, when our country is making new and untried demands upon our life-saving profession.

